# Biogeography and ecological functions of underestimated CPR and DPANN in acid mine drainage sediments

**DOI:** 10.1128/mbio.00705-25

**Published:** 2025-04-29

**Authors:** Sheng-Xuan Peng, Shao-Ming Gao, Zhi-Liang Lin, Zhen-Hao Luo, Si-Yu Zhang, Wen-Sheng Shu, Fangang Meng, Li-Nan Huang

**Affiliations:** 1 School of Life Sciences, Sun Yat-Sen University 26469 , Guangzhou, China; 2 School of Life Sciences, South China Normal University 12451, Guangzhou, Guangdong, China; 3 School of Environmental Science and Engineering, Sun Yat-Sen University 546480 , Guangzhou, Guangdong, China; Georgia Institute of Technology, Atlanta, Georgia, USA

**Keywords:** microbial ecology, ultramicrobacteria, DPANN archaea, biogeography, host-cell interactions

## Abstract

Recent genomic surveys have uncovered candidate phyla radiation (CPR) bacteria and DPANN archaea as major microbial dark matter lineages in various anoxic habitats. Despite their extraordinary diversity, the biogeographic patterns and ecological implications of these ultra-small and putatively symbiotic microorganisms have remained elusive. Here, we performed metagenomic sequencing on 90 geochemically diverse acid mine drainage sediments sampled across southeast China and recovered 282 CPR and 189 DPANN nonredundant metagenome-assembled genomes, which collectively account for up to 28.6% and 31.2% of the indigenous prokaryotic communities, respectively. We found that, remarkably, geographic distance represents the primary factor driving the large-scale ecological distribution of both CPR and DPANN organisms, followed by pH and Fe. Although both groups might be capable of iron reduction through a flavin-based extracellular electron transfer mechanism, significant differences are found in their metabolic capabilities (with complex carbon degradation and chitin degradation being more prevalent in CPR whereas fermentation and acetate production being enriched in DPANN), indicating potential niche differentiation. Predicted hosts are mainly *Acidobacteriota*, *Bacteroidota*, and *Proteobacteria* for CPR and *Thermoplasmatota* for DPANN, and extensive, unbalanced metabolic exchanges between these symbionts and putative hosts are displayed. Together, our results provide initial insights into the complex interplays between the two lineages and their physicochemical environments and host populations at a large geographic scale.

**IMPORTANCE:**

Candidate phyla radiation (CPR) bacteria and DPANN archaea constitute a significant fraction of Earth’s prokaryotic diversity. Despite their ubiquity and abundance, especially in anoxic habitats, we know little about the community patterns and ecological drivers of these ultra-small, putatively episymbiotic microorganisms across geographic ranges. This study is facilitated by a large collection of CPR and DPANN metagenome-assembled genomes recovered from the metagenomes of 90 sediments sampled from geochemically diverse acid mine drainage (AMD) environments across southeast China. Our comprehensive analyses have allowed first insights into the biogeographic patterns and functional differentiation of these major enigmatic prokaryotic groups in the AMD model system.

## INTRODUCTION

The extensive genomic exploration of natural environments has led to the discovery and functional inference of numerous major prokaryotic groups lacking pure culture representatives (1–3). Among these novel lineages, the candidate phyla radiation (CPR) bacteria and DPANN archaea have received particularly much attention as they constitute a major fraction of diversity in the tree of life (1–4) and have been detected in a wide variety of habitats including extreme environments (e.g., acid mine drainage [AMD] [5, 6], terrestrial geothermal springs [7], hypersaline habitats [8, 9], subsurface [2, 3, 10, 11], and deep sea [12, 13]). Cultured representatives of both groups are featured by ultra-small cells (average ~0.2 µm diameter [14]) and genome size (~250–2,000 kb [15]) and limited biosynthetic capabilities (1–3, 16), indicative of a symbiotic lifestyle (15, 17). Their close association with archaeal, bacterial, and even eukaryotic microbial hosts has later been supported by several enrichment studies (18–22). Notably, while deficient in major biosynthetic capacities, CPR and DPANN genomes encode genes involved in the carbon and nitrogen cycles, indicating their roles in driving biogeochemical cycles (15, 23). The continuous detection and in-depth genomic characterization of CPR and DPANN organisms in an expanding list of habitats have reshaped our understanding of prokaryotic diversity, metabolic functions, and evolution on the planet (11, 14, 15, 24).

Despite their extraordinarily high diversity and potential ecological and biogeochemical roles, the variation in the abundance and distribution of CPR and DPANN organisms remains largely unexplored (14). To date, most of the available near-complete CPR and DPANN genomes are extracted from groundwater environments, particularly those associated with the Crystal Geyser and Rifle aquifers metagenomes (2, 10, 25), and some previous studies have recovered and compared CPR genomes across multiple sampling sites (26–29). More recently, He et al. (14) sampled one agricultural and seven pristine groundwater sites in northern California sourced from multiple aquifers, documenting site-specific diversity of CPR bacteria and DPANN archaea likely driven by physiochemical conditions and host populations (14). Despite these pioneering works, the large-scale ecological ranges of CPR and DPANN organisms and potential drivers have not been systematically investigated.

Here, we report a comprehensive analysis of the biogeography and ecological functions of CPR and DPANN organisms in the extreme AMD environment. These drainage waters are primarily generated by the microbially mediated oxidative dissolution of mineral sulfides associated with mining activities, representing a significant environmental problem worldwide (30). The microorganisms in AMD are constrained by multiple environmental stresses, especially extreme acidity, toxic metals, and oligotrophy (31, 32). These low-diversity assemblages are ideal targets for the study of microbial evolution and ecological complexity in nature (33, 34) and have been subjected to extensive meta-omics analyses to resolve their diversity, community functions, and evolutionary dynamics (35–37). We have recently conducted metagenomic sampling of AMD sediments from 18 geographically separated (the maximum geographical distance between mining sites is 1,370 km) and geochemically diverse mine sites across southeast China, and the names, mineral types, and geographic and environmental information of the mining sites are provided in supplementary data (Table S1) (38). A total of 2,625 redundant metagenome-assembled genomes (MAGs) were recovered from the 90 metagenomic data sets. Notably, there were 282 CPR and 189 DPANN MAGs, which account for up to 28.6% and 31.2% of the corresponding prokaryotic communities. This provides a great opportunity to explore the biogeographic patterns of these unique microbial groups and the differentiation in their metabolic functions in the model AMD ecosystem. Previous studies found that contemporary environmental variation, especially pH, is the main factor explaining the overall community differences in AMD (39). However, as CPR and DPANN are potentially/most likely not free-living microorganisms and are physically associated with their prokaryotic hosts and local environment (they rely on the host for basic resources and typically encode numerous glycosyltransferases to attach to and regulate the local environment) (15), we hypothesized that their distribution patterns and ecological drivers are not determined by contemporary environmental variation but by geographic distance (i.e., dispersal limitation).

## RESULTS

### Abundance and diversity of CPR bacteria and DPANN archaea in AMD sediments

**Fig 1:**
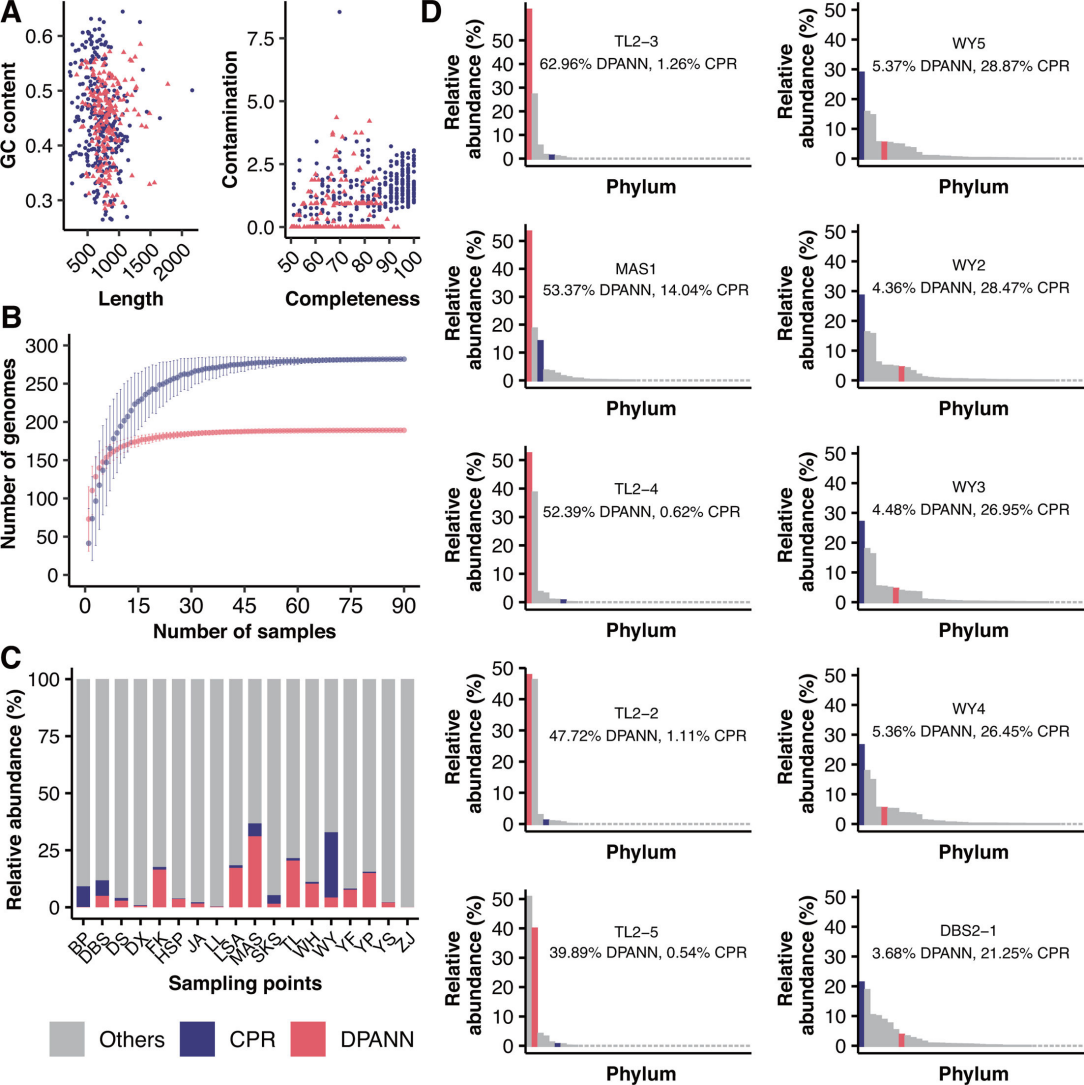
Overview and abundance of CPR and DPANN in AMD sediments. (**A**) Size, GC content, completeness, and contamination of CPR (blue) and DPANN (red) genomes. (**B**) Accumulation curves of CPR and DPANN genomes in the AMD sediments. Dots represent the average numbers of CPR and DPANN genomes for all combinations of a given number of samples, and error bars represent the range. (**C**) Relative abundances of genomes in the microbial communities, with CPR and DPANN highlighted and legend shared with other subfigures. (**D**) The abundance ranking curve of the rpS3 genes detected in the genome at each sampling site shows the five sampling sites with the highest relative abundance of CPR and DPANN, respectively.

Previously reconstructed MAGs from 90 AMD sediments across southeast China were screened for CPR and DPANN organisms (38, 40). A total of 282 CPR and 189 DPANN were identified. Consistent with previous research, the CPR and DPANN MAGs share the common feature of small genome size (14, 15). The smallest CPR genome size was 217 kb (79% completeness), while the smallest DPANN genome size was 326 kb (60% completeness; Fig. 1A). Overall, DPANN genomes were significantly larger than the CPR genomes, while the completeness of the latter was significantly higher (Table S2; Fig. S1). The accumulation curves of CPR and DPANN apparently approached saturation, indicating a relatively adequate sampling of both groups in the AMD sediments (Fig. 1B).

We used the relative abundances of genomes and rpS3 genes to examine and compare the community composition of the CPR and DPANN assemblages. Remarkably, relative abundances of the CPR and DPANN MAGs accounted for 0%–28.6% (average 3.4%) and 0%–31.2% (average 7.8%) of the total community abundance, respectively (Fig. 1C). The analysis of rpS3 genes revealed similarly high relative abundances of both groups, accounting for 0%–29% (average 3.4%) and 0%–63% (average 9.9%) of the total communities, respectively (Fig. 1D). Both approaches showed that CPR bacteria represent the most dominant taxa in the sediment communities of the DBS and WY mining areas, while DPANN archaea dominated the TL and MAS sediment communities (Fig. 1D; Fig. S2).

**Fig 2:**
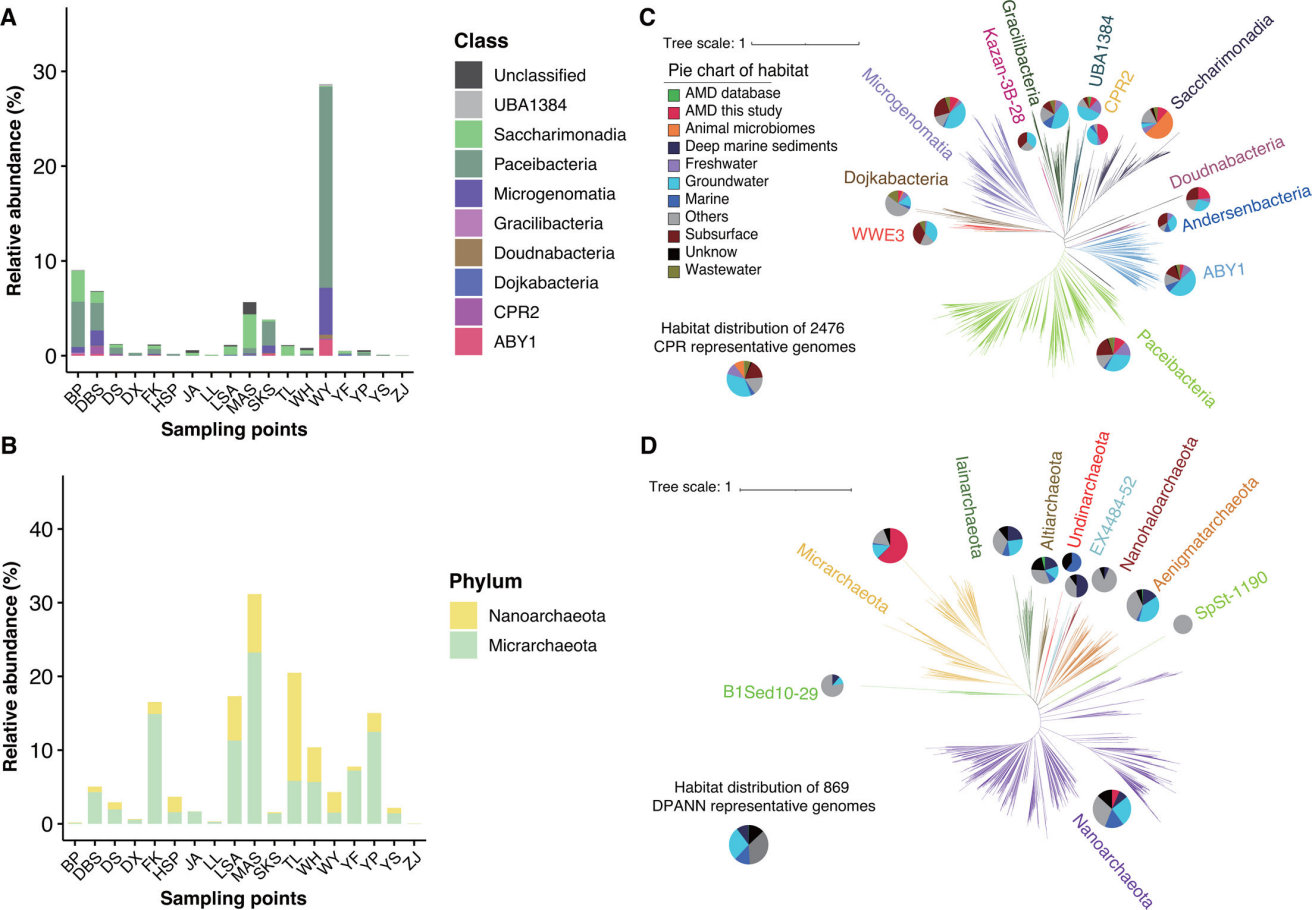
Distribution of CPR and DPANN in AMD and other environments. Genome relative abundances of class-level lineages within CPR (which was reclassified as a single phylum according to GTDB classification) (**A**) and DPANN (**B**) in all mining areas. Unrooted phylogenetic tree of the CPR (**C**) based on 15 concatenated ribosome proteins and of the DPANN (**D**) based on 14 concatenated ribosomal proteins (Fig. S3 and S4 show more readable trees). The tree scale is shown in the legend. The pie chart next to each lineage represents the environments from which the genomes in the lineage were recovered. The size of the habitat pie chart for CPR and DPANN, respectively, is proportional to the number of genomes (ln scale).

Among the recovered CPR and DPANN genomes, 9 of the 22 currently recognized class-level lineages of CPR, and 2 of the 11 currently recognized DPANN phylum-level lineages were identified in our data. Specifically, 82.6% of the CPR genomes were assigned to *Microgenomatia* (*n* = 49), *Saccharimonadia* (*n* = 54), and *Paceibacteria* (*n* = 130), and all DPANN genomes were affiliated with *Nanoarchaeota* (*n* = 38) and *Micrarchaeota* (*n* = 151; Fig. 2A and B). We further compared the CPR and DPANN MAGs with all the Genome Taxonomy Database (GTDB) representative genomes using average nucleotide identity (ANI) and found that 69 (24.5%) CPR genomes and 14 (7.4%) DPANN genomes could not be matched to the database (41) at the species level (>95% ANI [42]), indicating novel taxa in the AMD sediment community.

Thus, representative CPR (*n* = 2,476) and DPANN genomes (*n* = 869) from the GTDB database (41) were downloaded and combined with our genomes to construct phylogenetic trees. The results showed that most of the CPR (35.6%) and DPANN (42.3%) genomes in the public database were recovered from groundwater, marine, and deep-sea sediments, with only very few genomes (17 for CPR and 2 for DPANN) retrieved from AMD environments. Detected in our current study were CPR lineages (e.g., *Dojkabacteria*, *Doudnabacteria*, and *Gracilibacteria*) that have not or rarely been previously recovered from AMD systems (Fig. 2C and D). To identify new lineages at higher taxonomic levels in our data sets, we used relative evolutionary divergence to cluster all genomes into monophyletic groups (43). At the class level, we uncovered 10 novel CPR clades exclusively represented by MAGs from our study. Considerably more novel groups were identified at lower taxonomic ranks, with 32 clades identified at the family level and 158 clades identified at the genus level for CPR—exclusively represented by MAGs in this study—and for DPANN, 130 undiscovered clades were identified at the genus level (Fig. 2; Table S2 and S3), implicating AMD sediments as a hot spot for CPR and DPANN dark matter.

### Biogeographic patterns of CPR and DPANN assemblages

**Fig 3:**
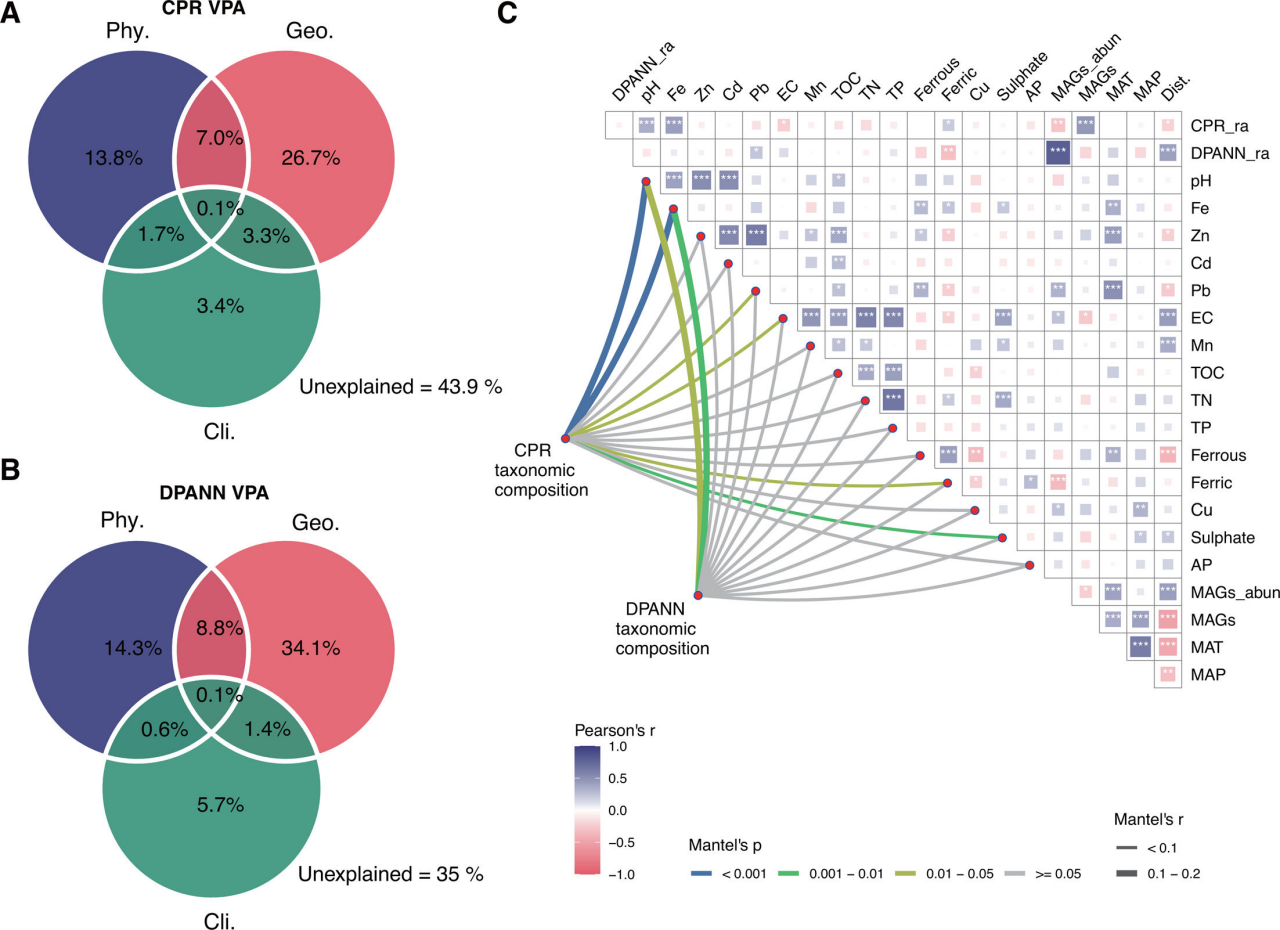
Biogeographic patterns of CPR and DPANN. VPA of the abundance variation of CPR (**A**) and DPANN (**B**) is explained by the comprehensive set containing physicochemical factors (Phy.), geographical distances (Geo.), and climatic variables (Cli.). The number represents the relative effect of each factor or a combination of factors. (**C**) Pairwise comparisons between various variables based on Pearson’s correlations (top right). The color gradient denotes Pearson’s correlation coefficient, and the star mark denotes statistical significance: *P < 0.05, **P < 0.01, and ***P < 0.001. Influence factors of CPR and DPANN taxonomic composition in partial Mantel tests under controlling geographical distance and climate variables (lower left). The edge width corresponds to Mantel’s *r* statistic for the corresponding distance correlations, and the edge color denotes the statistical significance. EC, electrical conductivity; Ferrous, ferrous iron; Ferric, ferric iron; TOC, total organic carbon; TN, total nitrogen; TP, total phosphorus; AP, available phosphorus; MAT, mean annual temperature; MAP, mean annual precipitation; Dist., distance from the equator; _ra, relative abundance; _abun, abundance.

A comprehensive data set including physicochemical factors, geographical locations, and climatic variables was used in combination with the CPR and DPANN MAGs collections to study the biogeographic patterns of these two groups in AMD sediments. Variance partitioning analysis (VPA) showed that geographical distances contributed most to the community variation of CPR (37.1%) and DPANN (44.4%), followed by physicochemical variables which collectively explain about 23% of the total variation for both CPR and DPANN assemblages (Fig. 3A and B). The distance-decay relationships (DDRs) also showed that the community similarity of CPR and DPANN decreased as the geographical distance increased (Fig. S5), indicating a potential influence of geographical isolation on the variations of the CPR and DPANN assemblages (Fig. S6).

Pearson correlation was further used to resolve the relationships between CPR/DPANN organisms and other biotic and abiotic factors (Fig. 3C). The results showed that pH (Pearson’s *r* = 0.35, *P* < 0.001), Fe (Pearson’s *r* = 0.42, *P* < 0.001), and prokaryotic richness (Pearson’s *r* = 0.46, *P* < 0.001) were most related to the relative abundance of CPR, while DPANN relative abundance was most associated with distance from the equator (Pearson’s *r* = 0.41, *P* < 0.001). To further examine the impact of independent physicochemical factors on the distribution of CPR and DPANN, a partial Mantel test was performed after controlling for geographical distance and climatic variables (Fig. 3C; Table S1). The results demonstrated that pH and Fe were most correlated with CPR/DPANN composition, while CPR composition was also related to EC and the total concentrations of ferric iron, Pb, and sulfate in the AMD sediments. Canonical-correlation analysis (CCA) of physicochemical factors and climate variables (Fig. S7) also showed that, for both CPR and DPANN, climate-related factors correlated with the first canonical variate, and the strongest correlations were mean annual temperature (MAT; 0.52 for CPR and 0.50 for DPANN) and Dist (0.40 for CPR and 0.37 for DPANN). In addition, the second canonical variate mainly consisted of pH (0.73 for CPR and 0.70 for DPANN) and iron (0.92 for CPR and 0.82 for DPANN).

### Metabolic potential of and differences between CPR and DPANN

**Fig 4:**
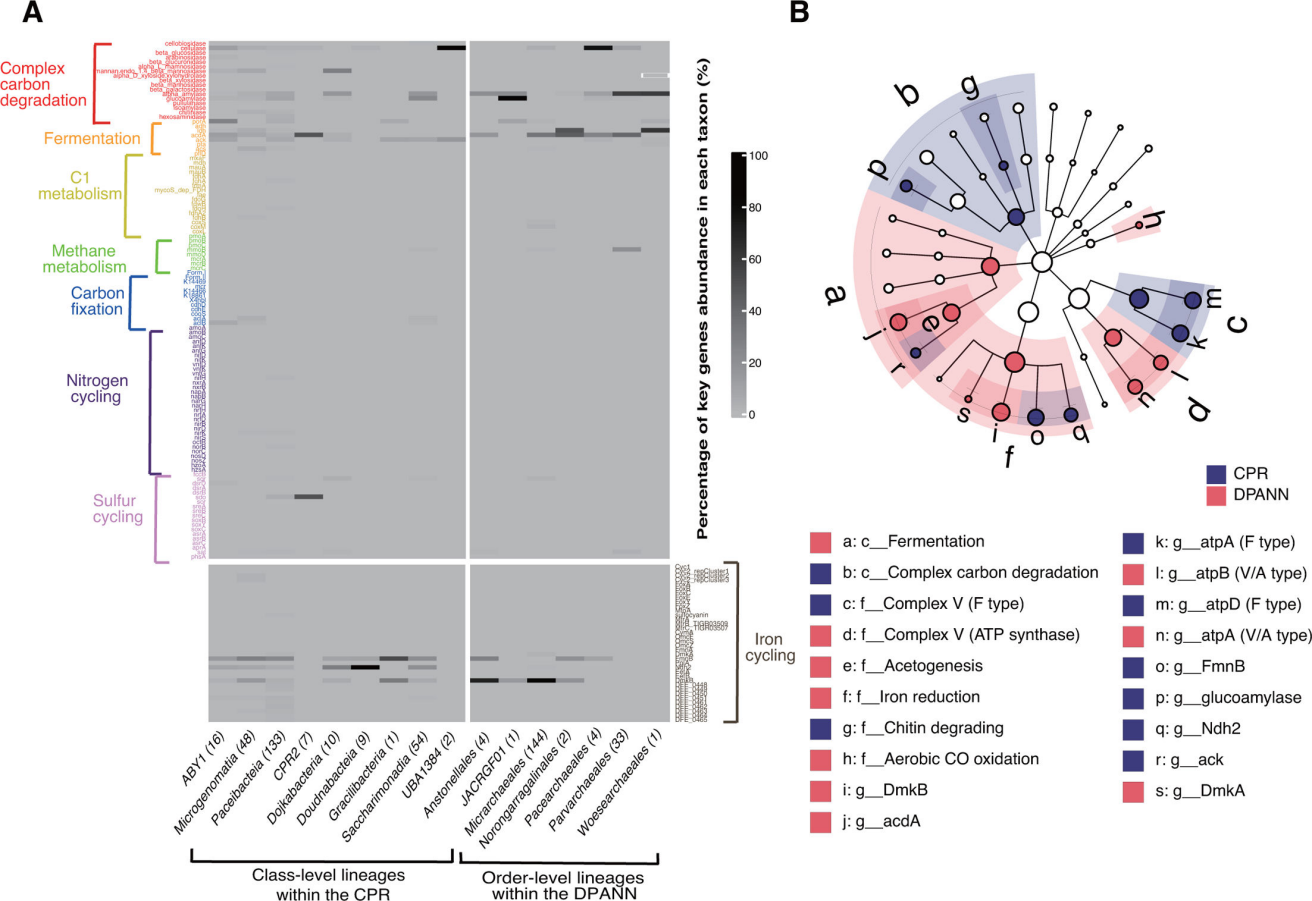
Profiles and biomarkers of CPR and DPANN metabolism. (**A**) Metabolic heatmaps of 282 CPR genomes and 189 DPANN genomes, with columns representing the class-level lineages of CPR and the order-level lineages of DPANN, and rows representing the relative abundance of key genes required for various metabolic and biosynthetic functions in the genomes of each taxonomic unit. (**B**) Biomarkers of metabolic capacity (gene [g], function [f], and category [c]). LEfSe cladogram showing CPR and DPANN biomarkers.

The overall high relative abundances and diversity of the recovered CPR and DPANN genomes merit an in-depth exploration of their metabolic potential and ecological functions in this understudied AMD environment. We specifically examined a curated set of key metabolic marker genes related to biogeochemical cycles in the genomes and evaluated their possible ecological roles based on gene sequence coverage on reads as abundance (see Materials and Methods). The results showed that both groups harbor almost no genes involved in methane metabolism, C1 metabolism, carbon fixation, and nitrogen and sulfur cycles. The few detected genes associated with these processes include the nitrogen-fixing gene *nifH*, nitrite reductase *nirK*, nitric oxide reductase *norB*, and sulfur dioxygenase *sdo* in the CPR genomes, as well as those encoding sulfide quinone oxidoreductase *sqr* and sulfate adenylyltransferase *sat* in the CPR and DPANN genomes (Fig. 4A; Table S4). In contrast, the CPR and DPANN genomes contain a higher abundance of genes involved in complex carbon degradation, fermentation, and iron reduction. Specifically, most of the identified carbon degradation and fermentation genes encode amylolytic enzymes, acetate kinase, acetyl-CoA synthetase, etc., and the iron-reducing functional genes are mainly involved in the flavin-based extracellular electron transfer (EET) mechanism (44) (Fig. 4A). In addition, we used FeGenie to identify iron-related genes in CPR and DPANN MAGs, providing more precise and accurate support for iron cycling function. The results revealed that both CPR and DPANN MAGs contained many genes involved in iron gene regulation and iron storage, indicating their potential roles in iron cycling (Table S5). Notably, three porin-cytochrome operons involved in iron reduction (previously identified in Geobacteraceae) were detected in CPR MAGs. In contrast, no other genes involved in iron reduction were found in DPANN (Table S5). We further used linear discriminant analysis effect size (LEfSe) analysis to identify metabolic biomarkers with significant differences between CPR and DPANN. While the CPR genomes are significantly enriched in genes or functions of F-type ATPase, complex carbon degradation, and chitin degradation, DPANN genomes are more enriched in V/A ATPase, fermentation, and acetate production (Fig. 4B).

Previous studies have demonstrated that both CPR and DPANN can degrade cellulose and starch into monomeric carbon, playing an important role in carbon degradation (3, 15). We used the CAZy database to identify specific glycoside hydrolases (GHs) to verify the significant differences in carbon degradation between the two lineages. The results showed that CPR genomes encoded α-amylases from GH13 (*n* = 1,909), GH57 (*n* = 148), GH119 (*n* = 5), and GH126 (*n* = 2), whereas DPANN genomes contained only GH13 (*n* = 594) and GH57 (*n* = 75) within this enzyme family. For cellulases, GH8 (*n* = 16) and GH5 (*n* = 461) were detected in CPR, while DPANN exclusively contained GH5 (*n* = 229; Table S6). Overall, including chitinases, CPR detected significantly greater numbers and diversity of GHs than DPANN, emphasizing its broader functional potential in carbon degradation. Interestingly, at the family level of CPR, UBA2103 and UBA4665_A contain the most α-amylase GHs (UBA2103 has 163, and UBA4665_A has 342) and chitinases GHs (UBA2103 has 114, and UBA4665_A has 134), accounting for approximately 25% of these two GHs in CPR. We next examined biosynthetic gene clusters (BGCs) in the CPR and DPANN genomes to assess their biosynthetic capabilities. A total of 37 putative BGC regions (21 in CPR and 16 in DPANN) were identified using AntiSMASH (v7.1) (45), among which 16 and 9 ribosomally synthesized and post-translationally modified peptides (RiPPs)-related BGCs were encoded by CPR and DPANN, respectively (Table S7). The RiPPs-related BGCs of CPR mainly encode YcaO proteins (*n* = 5), which mediate the thioamidation (a posttranslational modification) of methyl-coenzyme M reductase (MCR) (46). Additionally, a methanogenesis marker protein 1 was detected in CPR. These genes are involved in thioamidation, and the absence of this modification in MCR might be particularly detrimental (46, 47). Thus, we speculate that the CPR organisms may help methanogenic and methanotrophic archaea anaerobically produce and consume methane.

### Host prediction and interactions

**Fig 5:**
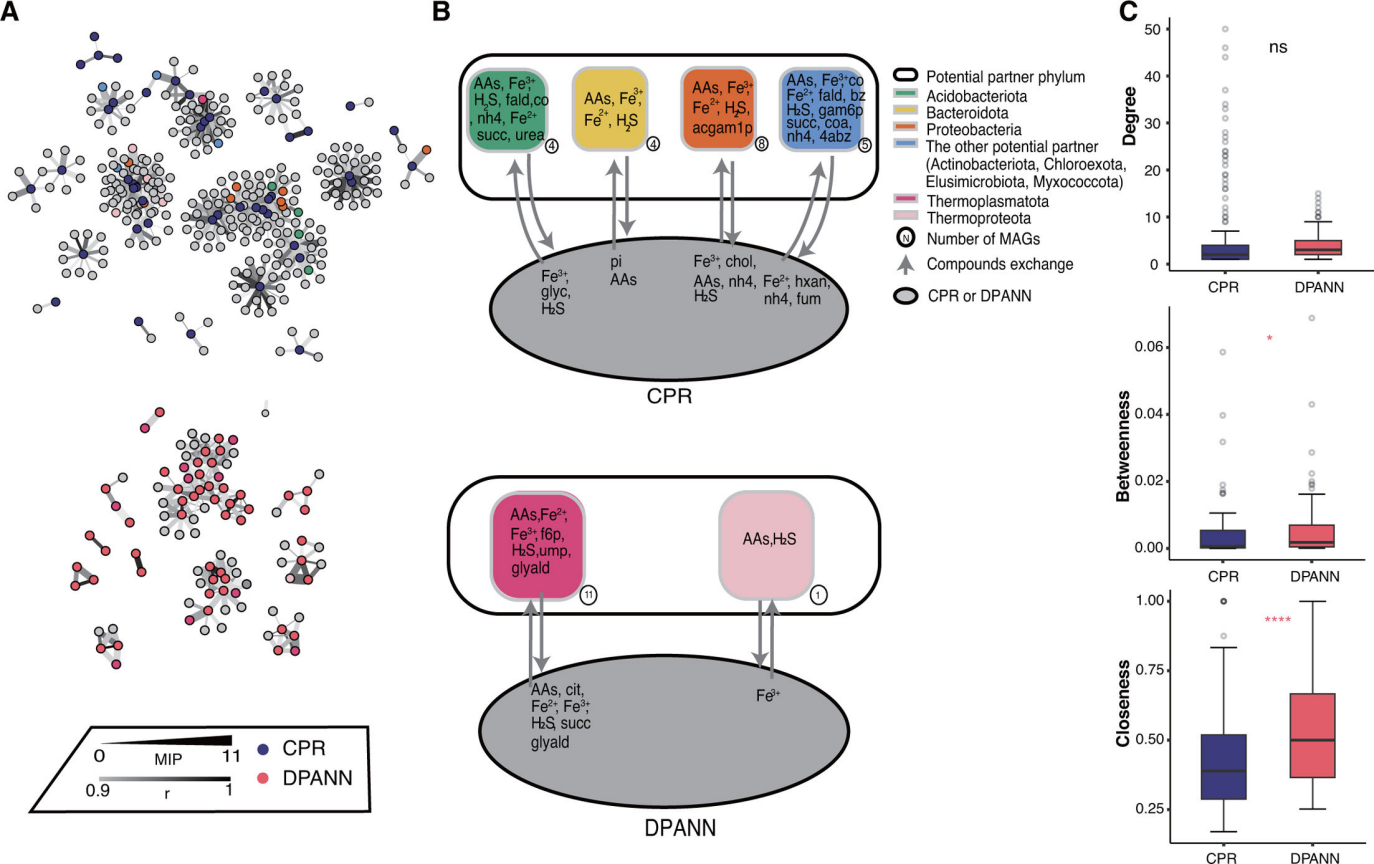
Co-abundance patterns and host interactions of CPR and DPANN. (**A**) Microbial community co-occurrence network of CPR (above) and DPANN (below) in the AMD sediment samples. Each node represents a genome (average relative abundance > 0.1%), and each edge represents a strong and significant correlation between two points (Pearson’s |*r|* > 0.9, *P* < 0.05). Nodes classified as CPR are marked in red and DPANN in blue. The nodes and edges connected to CPR and DPANN are marked in different colors to represent their phylum-level classification levels, and the colors are the same as in the legend for panel **B**. (**B**) Interaction patterns of CPR and DPANN with their putative hosts. Putative hosts are grouped at the phylum level. The number of MAGs in each group is shown in the lower right corner. The black arrow represents compounds exchanged. Specific metabolic exchange information is provided in Table S9. The compounds in the group were derived by SMETANA and verified with Kyoto Encyclopedia of Genes and Genomes (KEGG) Mapper. AAs, amino acids; 4abz, 4-aminobenzoate; acgam1p, N-acetyl-D-glucosamine 1-phosphate; bz, benzoate; CO, carbon monoxide; coa, coenzyme A; f6p, D-fructose 6-phosphate; fald, formaldehyde; gam6p, D-glucosamine 6-phosphate; glyald, D-glyceraldehyde; nh4, ammonium; succ, succinate; urea, urea CH_4_N_2_O. (**C**) Comparison of node-level topological characteristics (degree, betweenness centrality, and closeness centrality) of CPR and DPANN and the Wilcoxon rank sum test. ns, 0.05 < *P*; *, 0.01 < *P* < 0.05; **, 0.001 < *P* < 0.01; ***, 0.0001 < *P* < 0.001; and ****, *P* < 0.0001.

Considering the symbiotic lifestyle of most CPR and DAPNN organisms, we next sought to predict their hosts which would impact their distribution and metabolic functions. Both genome-scale metabolic models and microbial co-abundance networks were used to predict the putative hosts (see Materials and Methods). We calculated metabolic interaction potential (MIP) scores to show the number of essential nutrients shared by the species pair through metabolic exchange. It was observed that the number of interacting species for both CPR and DPANN increases first and then decays exponentially with the increase in the MIP score for them (Table S8; Fig. S8). This MIP score was thus set as a threshold to distinguish the potential cooperation between CPR/DPANN and their hosts. We next performed a co-abundance network analysis, focusing on positive correlations with edge confidence above a cutoff of 0.9 only. The results showed that the CPR MAGs have co-abundance patterns with 681 other genomes and DPANN with 196 other genomes (Fig. 5A). Finally, we identified those pairs that showed relatively high interaction potential and had co-abundance patterns as CPR/DPANN and their putative hosts. Specifically, the predicted hosts of CPR were mainly *Acidobacteriota* (*n* = 4), *Bacteroidota* (*n* = 4), and *Proteobacteria* (*n* = 8), while those of DPANN were mainly *Thermoplasmatota* (*n* = 11). At the genus level, the predicted hosts of CPR were mainly *Thiomonas* (*n* = 3), while those of DPANN were mainly *Cuniculiplasma* (*n* = 2) and UBA509 (*n* = 2).

We further investigated specific metabolite exchanges between CPR/DPANN organisms and their hosts (Fig. 5B). It was found that both CPR and DPANN mainly obtain various amino acids and hydrogen sulfide from their hosts (Table S9). Additionally, CPR and DPANN organisms may obtain some metabolic intermediates such as formaldehyde, succinate, benzoate, D-fructose 6-phosphate, and D-glyceraldehyde from their hosts to feed themselves, and they mainly provide ferrous and ferric iron to their hosts. The smetana scores (a measure of certainty on metabolic exchange) (48) were then compared when CPR/DPANN or their hosts acted as receivers. The results showed that the smetana score was significantly higher when CPR and DPANN organisms acted as the receiver (Table S9 and Fig. S9), indicating that both groups obtained more compounds from their hosts but contributed less. However, when comparing the node-level topological features that can be used to identify keystone taxa (49) in two co-abundance subnetworks of CPR/DPANN (Fig. 5A), the betweenness and closeness centrality of the DPANN subnetwork were significantly higher than those of the CPR subnetwork. This result suggests that DPANN has greater importance in maintaining microbial community structure and function (Fig. 5C). Other network-level topological characteristics also showed that the DPANN subnetwork displayed significantly higher clustering coefficient and graph density but lower average path length and diameter than the CPR subnetwork (Table S10), implying stronger interconnectivity between DPANN organisms and accompanied species than CPR.

### Case study of iron reduction

**Fig 6:**
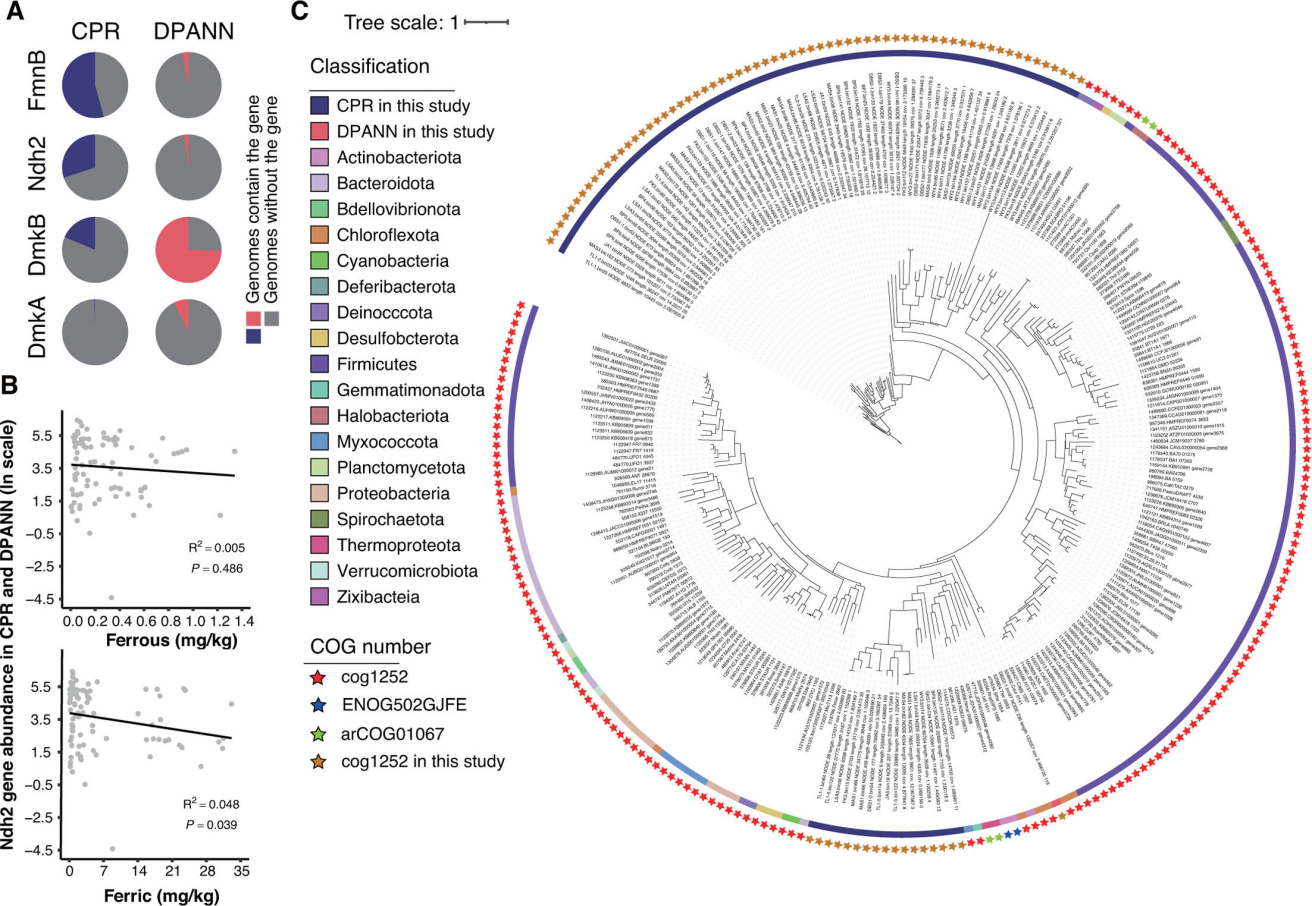
Analysis of iron reduction-related genes in the CPR and DPANN genomes. (**A**) Pie charts of the percentages of CPR and DPANN genomes containing the flavin-based EET mechanism genes. Genes not identified in the genomes of both lineages are not shown. (**B**) Linear regression relationship between the total abundance of CPR and DPANN Ndh2 genes and the concentration of ferrous (top) and ferric (bottom) iron. The statistical test used was two tailed. (**C**) Maximum-likelihood phylogenetic tree with Ndh2 genes from CPR and DPANN compared to homologs found in eggNOG v5.0.0 database, colored according to the phylum level classification of the origin of the homologs (the color ring). The Clusters of Orthologous Genes (COG) numbers are indicated by a star.

As iron and sulfur cycling are central to the functioning of AMD ecosystems, and the detected iron-reduction genes are mainly associated with the flavin-based EET mechanism, we further analyzed these functional genes from CPR and DPANN in detail. The EET mechanism consists of eight genes (*Ndh2*, *DmkB*, *DmkA*, *EetB*, *EetA*, *FmnA*, *FmnB,* and *PplA*) that deliver electrons to iron or electrodes. Specifically, the *Ndh2* gene plays a role in the initiation of the EET process by transferring electrons from NAD to demethylmenaquinone (DMK) synthesized by *DmkA* and *DmkB*. The electrons are then transferred to flavin mononucleotide (FMN) groups on *PplA* modified by *FmnA* and *FmnB* or on free flavin shuttles (44). We found that both CPR and DPANN contain genes encoding *FmnB*, *Ndh2*, *DmkA*, and *DmkB*. Specifically, *FmnB* and *DmkB* were identified in more than half of the CPR (54%) and DPANN (74%) genomes, respectively (Fig. 6A), indicating that iron reduction genes are very prevalent in these lineages. In supporting this, linear regression analysis revealed that the abundance of *Ndh2* genes in CPR and DPANN was significantly negatively correlated with the total concentrations of ferric iron instead of ferrous iron in the AMD sediments (Fig. 6B).

We then compared the *Ndh2* genes identified in our CPR/DPANN genomes with the eggnog database (50) and found that three COG members (cog1252, ENOG502GJFE, and arCOG01067) were matched. We recruited 178 homologs with the highest similarity (Table S11) and merged them with our CPR/DPANN *Ndh2* genes (82 in CPR and 1 in DPANN) to reconstruct a phylogenetic tree (Fig. 6C). The result showed that the *Ndh2* genes designated as cog1252, which include all *Ndh2* genes from our CPR and DPANN genomes, accounted for the majority of the homologs. The genes designated as ENOG502GJFE originated from *Actinobacteriota*, while those designated as arCOG01067 were from *Halobacteriota*. In addition, the *Ndh2* gene cluster of CPR was divided into two branches, with one branch clustered with cog1252 and the other clustered with the *Ndh2* genes from DPANN and other archaea. An additional analysis of community-level horizontal gene transfer (HGT) events in our data (see Materials and Methods) showed that the *Ndh2* genes in the AMD sediment communities were not involved in HGT. Thus, we speculate that there were ancient HGT events for the CPR *Ndh2* genes clustered with archaea, as well as a potential cross-domain symbiosis between CPR and archaea (Table S12 and S13).

## DISCUSSION

Early metagenomic sequencing has enabled the discovery of Archaeal Richmond Mine acidophilic nanoorganisms (ARMAN; *Micrarchaeota* and *Parvarchaeota*) in the AMD biofilms from the Richmond Mine (5, 51). These archaea are among the earliest reported DPANN lineages, and their cell sizes are close to the theoretical lower limit for life (22, 51). The massive recovery from our AMD sediments of novel CPR and DPANN genomes is somewhat not unexpected given the putative anaerobic lifestyle of most of these atypical prokaryotes, and previous genomic surveys of the AMD ecosystem have largely been biased toward the AMD solution and biofilms (35–37). The availability of these MAG collections from a wide geochemical and geographic range motivated us to investigate the biogeographic patterns of the CPR and DPANN organisms, thus filling a major knowledge gap of these lesser-known prokaryotes in general.

Our analyses revealed that the abundance variation and community composition of CPR and DPANN organisms are most related to geographic distances (Fig. 3A and B; Fig. S5). This is inconsistent with several previous microbial biogeography studies of AMD, which suggest that contemporary environmental variation, rather than geographic distance, primarily explains community differences (39). This finding supports our hypothesis and implies that processes underlying patterns of these ultra-small prokaryotic assemblages may be distinct from those for the overall communities. This may be due to the fact that CPR and DPANN are typically non-free-living organisms that are physically attached to their host populations (16, 52). In addition, CPR and DPANN organisms have been documented to encode abundant glycosyltransferases (3, 53), which may allow the production of substantial amounts of saccharides, polysaccharides, or glycoproteins to participate in the attachment and regulation of the local environment around the host cell surface (15, 53). This feature would limit their dispersal capability, ultimately leading to spatial isolation by distance. Future investigations examining large-scale distribution patterns of CPR and DPANN in other habitats may provide additional evidence for this assumption. On the other hand, our results also indicated that geochemical parameters, especially pH and iron, have an influence on community patterns of CPR and DPANN organisms. Interestingly, both variables have been found as the main environmental factors shaping variations in AMD communities in previous studies. For example, pH can largely predict the diversity patterns of 59 AMD microbial communities in southeast China (39), and iron gradients between sampling stations along the Rio Tinto River are responsible for community differences (54). These findings are reasonable as both extreme acidity and elevated heavy metal concentrations are distinctive features of AMD ecosystems and thus may have a strong selective effect on the microbial inhabitants including CPR and DPANN.

Despite their reduced genomes lacking major biosynthetic capabilities, recent studies have suggested the role of CPR and DPANN in the carbon cycle (3, 55). Our analysis of the CPR and DPANN MAGs from the AMD sediments supports this finding, unveiling their metabolic potential for the degradation of cellulose and starch into monomeric carbon and the production of carbon compounds such as acetate, lactic acid, ethanol, and formate through fermentation pathways to support the growth of other community members (Fig. 4A). Notably, a previous comparative analysis of approximately 1,000 genomes reconstructed from several metagenomics-based studies has found striking similarities in the gene presence/absence patterns of CPR and DPANN (15), underscoring the consistency of their metabolic content. Our comprehensive analysis of the AMD sediment MAGs, however, uncovered major differences in the metabolic capabilities between CPR and DPANN, especially in the carbon degradation processes. Specifically, CPR bacteria mainly perform starch (GH13, GH57, GH119, and GH126) and chitin (GH18, GH19, GH23, and GH48) degradation, whereas DPANN archaea mainly perform fermentation processes such as acetic acid generation (Fig. 4B). Thus, CPR and DPANN organisms may undergo metabolic differentiation in the oligotrophic AMD environments to avoid competition for the limited resources. Additionally, our study also found that the CPR and DPANN genomes contain only a few genes involved in denitrification, sulfur and sulfide oxidation, and sulfate reduction pathways. As these pathways are largely incomplete, we anticipate that these genes may be retained by the CPR and DPANN for the exchange of metabolic intermediates with their hosts (Fig. 4A).

Multiple studies have previously used a co-abundance pattern to predict the hosts of CPR and DPANN organisms (11, 28, 56, 57). Our study moved a step further by integrating metabolic model reconstruction with this approach. The metabolic interactions predicted by the model suggested that both groups rely on their hosts to provide various amino acids. This result is supported by several recent investigations reporting that, in order to reduce individual metabolic burden, CPR and DPANN organisms often lack the necessary pathways to synthesize amino acids, especially those that are expensive in terms of energy cost to synthesize (3, 15, 23, 58). In addition, some specific compound exchanges, such as formaldehyde, 4-aminobenzoate, urea, etc., seem to have not been recognized in previous studies, expanding our knowledge of the metabolic interactions of CPR/DPANN with their hosts. Such knowledge may aid future efforts to bring these uncultured prokaryotes to cultivation (e.g., enrichment cultures). It should be noted that our analysis did not account for potential cross-domain symbioses involving CPR and DPANN. Such a phenomenon has recently been demonstrated by a case of symbiosis between CPR and methanogenic archaea (24).

We identified a variety of flavin-based EET iron-reducing genes in the CPR and DPANN genomes. However, none of these MAGs encodes a complete set of EET genes, with *EetA*, *EetB*, *FmnA*, and *PplA* typically missing, suggesting that the respective proteins may catalyze different reactions in a different metabolic context. However, previous studies on this EET mechanism found that electrons are transferred from NAD to DMK and then to the FMN group on *PplA* or free flavin shuttles (44). This means that the *PplA*, *FmnA*, and *FmnB* genes in the EET mechanism may be non-essential if flavins are available in the environment for utilization. Therefore, further studies have speculated that the flavin-based EET mechanism might be different in microorganisms, or proteins performing alternative functions could be identified. Taken together, even if incomplete, we speculate that these CPR and DPANN organisms in AMD sediments might be capable of this unique EET. Additionally, considering that there are many other iron-reducing bacteria in the AMD community (59), the weak but significant negative correlation between the total abundance of Ndh2 genes in CPR and DPANN genomes and the concentration of ferric iron in the AMD sediments also partially supported this speculation. As previous omics surveys have largely ignored CPR and DPANN organisms and thus their unusual iron-reducing capabilities *in situ* (34, 59), this unique electron transfer mechanism may complement our understanding of iron transformation in AMD environments. This complement is important because AMD ecosystems are largely driven by iron and sulfur cycles.

Our comprehensive analyses have provided foundational observations of the patterns and drivers of the large-scale ecological distribution of CPR and DPANN microorganisms. Although viruses (bacteriophages) may exert top-down controls on prokaryotic abundances and community structure in natural microbial assemblages (60–63), the extent to which viruses contribute to the distribution and dynamics of CPR and DPANN organisms remains unknown. This issue could be challenging to address, as the interplays between these putatively symbiotic microorganisms, their hosts, and their respective viruses would be inherently complex. Meanwhile, the high abundances of CPR and/or DPANN in many of the analyzed AMD sediments implicate their potentially important ecological roles. Our comparative genomic analyses have dissected their metabolic capabilities, including involvement in iron reduction, and uncovered significant functional differentiation, especially in the carbon cycle, between the two groups. Future efforts are needed to resolve the adaptive benefits and fitness consequences of such metabolic differentiation and the evolutionary processes underlying it. As demonstrated in the present study, AMD sediments, which may contain abundant and diverse CPR and DPANN assemblages, may serve as models for exploring the unique facets of the biology and ecology of these ultra-small, enigmatic microorganisms.

## MATERIALS AND METHODS

In previous studies from our laboratory, AMD sediments were collected from 18 geochemically and mineralogically diverse mining areas across southeast China (22.96°−31.68°N and 105.73°−118.63°E) from August to October 2017. For each sample, surface sediment (at the top 10 cm) was collected from the center of the AMD pool or about 1 m away from the edge of the pool (38, 40). Sediments were kept in an icebox and transported to the laboratory, where they were well mixed and divided into two fractions: one for total community genomic DNA extraction (stored at −80°C prior to extraction) and the other (air dried) for physicochemical measurements (40). Gao et al. used the MiSeq Reagent Kit v3 on an Illumina MiSeq platform for sequencing from both ends (150 bp, paired-end reads). This generated a total of ~7 Tb of metagenomic raw read data. Metagenomic reads were quality filtered and trimmed using an in-house Perl script and assembled using SPAdes v3.14.1 and kmers of 21, 33, 55, 77, 99, and 127 under the “--meta” mode (64). Binning was performed from 90 sediment metagenomic assemblies using MetaBAT v2.12.1 (65), MaxBin v2.2.2 (66), Abawaca v1.00 (2), and Concoct v0.4.0 (67) with default parameters, considering tetranucleotide frequencies, scaffolds coverage, and GC content. The resulting bins were then merged using DASTool v1.1.2 (68) and further manually curated using RefineM v0.0.24 (69) to obtain 2,625 MAGs (genome completeness ≥ 50% and contamination < 10%) (38). Additionally, as the representative genome of each population, their calculated value of “completeness − 4× contamination” as genome quality was the highest among the populations (38). An associated comprehensive data set of physicochemical properties, geographical distances, and climatic variables has been published in two previous articles (38, 40). Specifically, the physicochemical parameters measured include pH, EC, total nitrogen, total phosphorus, total organic carbon, irons, heavy metals (Fe^2+^, Fe^3+^, Fe, Pb, Zn, Cu, Cd, and Mn), and sulfate. The geographic and climate data include longitude and latitude, and MAT, and mean annual precipitation, respectively.

### Genomic and functional annotation

The 2,625 prokaryotic genomes were classified using the classify_wf command of the genome taxonomy database (GTDB-Tk v2.1.0) (70), and genomes of CPR bacteria and DPANN archaea were identified. We then computed the ANI of the CPR and DPANN genomes to all GTDB-Tk representative genomes using the ani_rep command (70). The completeness and contamination of genome bins were assessed using CheckM v1.2.2 (71) with default parameters, except those assigned as CPR. These CPR genomes were estimated for completeness using a set of 43 marker genes (2) and were estimated for contamination using the universal set of bacterial marker genes. Genes of CPR and DPANN genomes were predicted by Prodigal v2.6.3 (72). Iron reduction genes in the CPR and DPANN MAGs were identified using FeGenie (73), and carbohydrate-active enzymes in CPR genomes were identified using the CAZy database (74).

The program METABOLIC (v.4.0) (75) was used to search predicted genes against a curated set of KEGG, TIGRfam, Pfam, and custom hidden Markov models (HMM) profiles corresponding to key marker genes. Raw reads were then mapped to sequences designated as metabolic marker genes using Bowtie2 (v 2.5.1) (76), and gene abundance was represented by the coverage. METABOLIC was also used to evaluate the metabolic capacities of the genome by computing the completeness of KEGG modules for key biogeochemical cycling processes. Specifically, the presence/absence of key genes was determined by profiling against a custom set of HMM, and the combination of key genes (defined by the KEGG database) determines the presence/absence pattern of each function in the relevant KEGG module. Finally, a given KEGG module was considered to be present if genes identified for >75% of the reactions in the module were detected.

### Secondary metabolism

BGCs were identified from the CPR and DPANN genomes using antiSMASH (v7.1.0) (45) with the following parameters: --fullhmmer --genefinding-tool prodigal-m --cb-general --cb-subclusters --pfam2go. Only BGCs on contigs of >5 kb were considered. Specific BGC families were identified using experimentally validated BGCs in the MIBiG database 2.0 (77) by BiG-SCAPE (v1.1.5) (78). To analyze the biochemistry of BGCs, BiG-SCAPE divides BGC families into six major classes: RiPPs, polyketide synthases (PKSs), nonribosomal synthetic peptides (NRPS), polyketide-nonribosomal peptide combinations (PKS-NRPS hybrids), terpenes, and others. Finally, BGCs were compared to the eggNOG v5.0.0 database (50) to identify homologs with an *E*-value cutoff of 1 × 10^−3^.

### Phylogenetic classification

For phylogenetic analysis, ribosomal proteins of the CPR and DPANN genomes recovered from our data sets were concatenated with those of the CPR/DPANN genomes in the GTDB database (41). Representative CPR and DPANN genomes in the GTDB database were selected, and their Genome Collections Accession (GCA) numbers were downloaded. The NCBI’s data format command-line tool was used to download a detailed data report for each GCA number, and the corresponding habitat information was then manually counted. The concatenated ribosomal protein set for bacteria includes 15 proteins (L2, L3, L4, L5, L6, L14, L15, L18, L22, L24, S3, S8, S10, S17, and S19), while the archaeal set includes 14 proteins (bacterial set without S10). We integrated the HMM files of the above ribosomal protein genes into the AMPHORA2 software (79) and used MarkerScanner.pl to pick up the ribosomal protein genes of CPR and DPANN genomes with the *e*-value of 1e-7 and the matching area of the HMM larger than 70%. All individual ribosomal proteins were then concatenated together and aligned with MAFFT (v7.520) (80) after the excision of unconserved regions using trimAl (v1.4.rev22) (81). Phylogenetic analysis was performed using iqtree2 (v2.1.3) (82) with the parameters of “-m MFP -B 1000 --bnni.”

For the analysis of the key EET mechanism gene *Ndh2* (44), predicted *Ndh2* genes in the CPR and DPANN genomes were assigned to the eggNOG v5.0.0 database (50) (BLASTp, threshold of 50 for bit score and 10^−5^ for *E*-value) to recruit homologous sequences (up to five for each *Ndh2* gene). The *Ndh2* genes were then aligned with MAFFT (v7.520) (80) and filtered by TrimAL (v1.4.rev22) (81) to remove columns composed of more than 95% gaps. Finally, a phylogenetic tree was constructed using iqtree2 (v2.1.3, iqtree2 -s <alignmentfile> m MFP -B 1000 --bnni) (82). For the identification of community-level HGT, MetaCHIP (83) was used to detect HGT events in the phylum-level lineages of the AMD sediment communities. The results were scanned specifically for *Ndh2* genes to verify whether they were involved in HGT events.

### Statistical analyses

Statistical analyses were performed using various packages in the statistical program R v4.3.2. LEfSe analysis was performed using the microeco package to identify genes, functions, and categories with significant quantitative differences between the CPR and DPANN groups. Biological and abiotic matrices were normalized by the “Hellinger” and “normalize” methods, respectively, using the “decostand” function in vegan v2.6-4. Bray-Curtis differences were used to display distances between CPR and DPANN community structures, while Euclidean distances were calculated using environmental variables (vegan v2.6-4). The varpart function in the vegan package was used to quantitatively calculate the impact of geographical distance, environmental variables, and climate factors on CPR and DPANN community variations. Pearson correlations were performed using the “rcorr” function in Hmisc v5.1-1 (999 permutations) to assess the relationship between CPR and DPANN populations, functions, physicochemical variables, and climatic factors in all samples. Partial Mantel tests were performed by controlling for the effects of geographical distance and climatic factors to reveal how environmental factors affect the correlation between dissimilarity matrices. To examine how the local spatial organization of CPR and DPANN communities varies within and between different AMD sites, CCA (vegan v2.6-4) was used. DDR rates were calculated as the slope of a linear least squares regression of the relationship between log10-transformed geographic distance and similarity in the CPR/DPANN taxonomic and functional community composition.

### Host prediction analysis

Putative hosts of CPR and DPANN were predicted based on co-abundance patterns and metabolic models. Specifically, after removing the rare taxa, the rcorr function in the *R* package was used to calculate the Pearson correlations of pairwise genome abundances. When *P* < 0.5 and *R* > 0.9, these pairs were considered to have co-abundance patterns, and pairs containing CPR and DPANN were filtered out. Metabolic models for the selected pairs were subsequently established using CarveMe (84). The global model of SMETANA (48) was then used to calculate the MIP, and pairs with high MIP were filtered out as putative host pairs for CPR/DPANN. Specific metabolite exchanges between metabolic models were further calculated using the detailed mode of SMETANA, considering only compounds with a SMETANA score greater than or equal to 0.1. The pathways associated with these compounds were then manually inspected in the reconstructed network of the corresponding MAG using KEGG Mapper. A compound was finally considered to be exchanged between the two MAGs only if the donor and the receiver simultaneously possessed the genes for synthesizing and utilizing the compound, respectively.

## Data Availability

Raw reads of metagenomes and all assembled prokaryotic population genomes have been deposited in NCBI BioProject database under accession code PRJNA666025. eggNOG database is available at http://eggnog5.embl.de/download/eggnog_5.0. The in-house Perl scripts, R scripts, and relevant data used to generate figures of this study are provided with this paper and publicly available on GitHub at https://github.com/eco-pengsx/CPR-DPANN-biogeography.

## SUPPLEMENTAL MATERIAL

Supplemental figures mbio.00705-25-s0001.docx Fig. S1 to S9.

Supplemental tables, part 1 mbio.00705-25-s0002.xlsx Tables S1 to S6.

Supplemental tables, part 2 mbio.00705-25-s0003.xlsx Tables S7 to S13.

ASM does not own the copyrights to Supplemental Material that may be linked to, or accessed through, an article. The authors have granted ASM a non-exclusive, world-wide license to publish the Supplemental Material files. Please contact the corresponding author directly for reuse.
