# Supplementary material for: Biogeography and ecological functions of underestimated CPR and DPANN in acid mine drainage sediments
Source: mBio. 2025 Apr 29;16(6):e00705-25. doi: 10.1128/mbio.00705-25 (PMC12153355; doi:10.1128/mbio.00705-25)
Supplement: Supplemental figures — Fig. S1 to S9. [file mbio.00705-25-s0001.docx]

**Supplementary Figure**


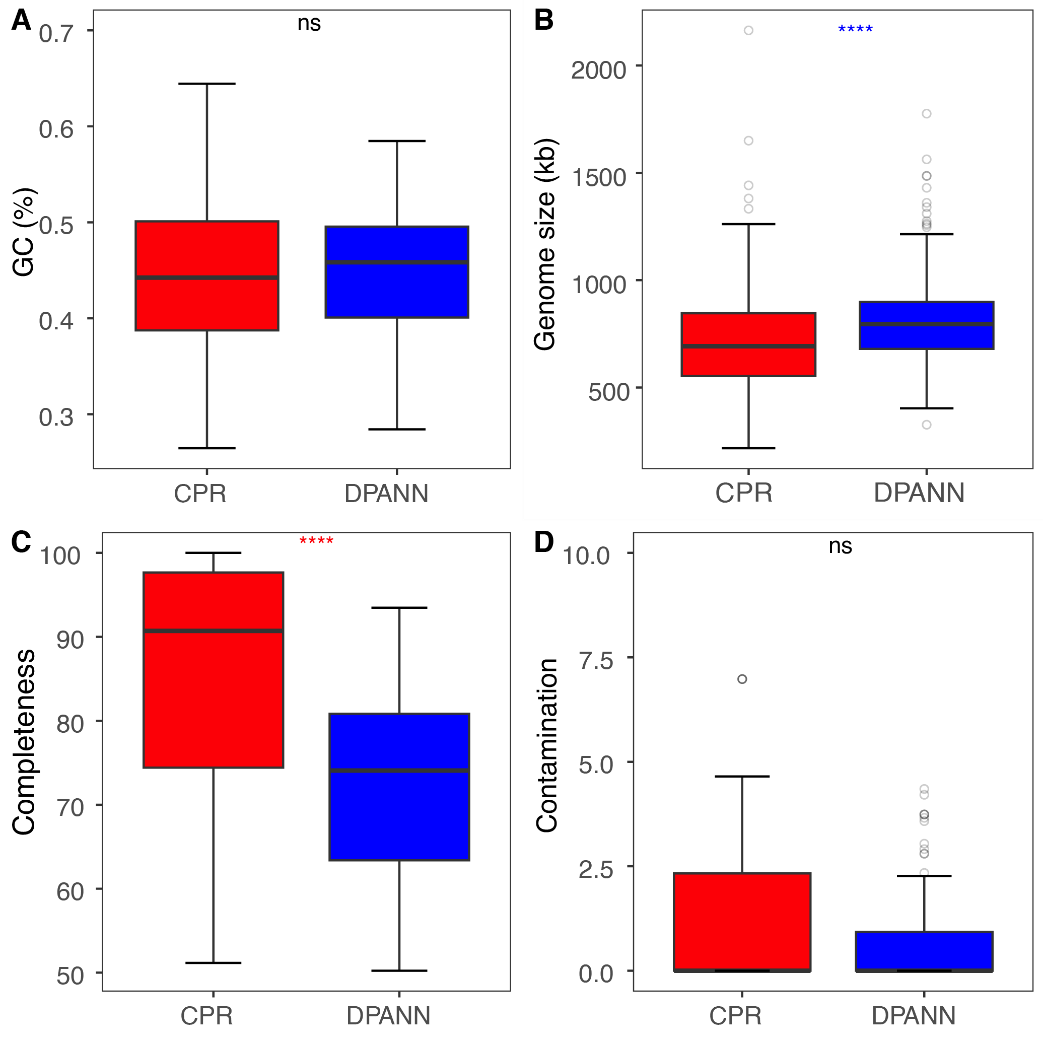


**Fig S1. Comparison of some genome contents between CPR and DPANN using the Wilcoxon rank sum test.** GC content (A), genome size (B), completeness (C) and contamination (D) were compared respectively. ns 0.05 < P, * 0.01 < P < 0.05, ** 0.001 < P < 0.01, *** 0.0001 < P < 0.001 and **** P < 0.0001.


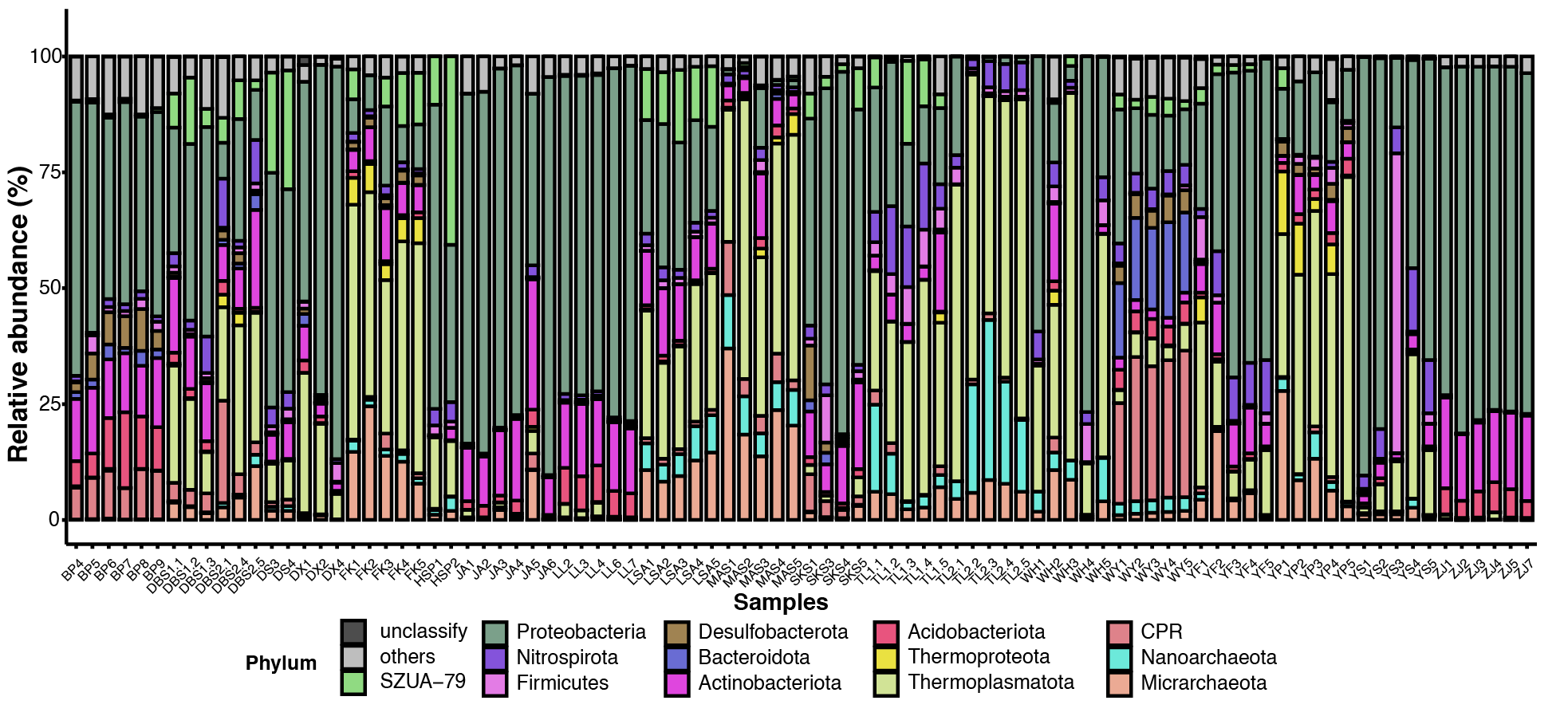


**Fig S2.** **Relative genome abundance by phylum-level lineage in 90 AMD sediment samples.** Rare species in the community are represented by others (dark yellow), Gray represents unclassified genome relative abundance.

**
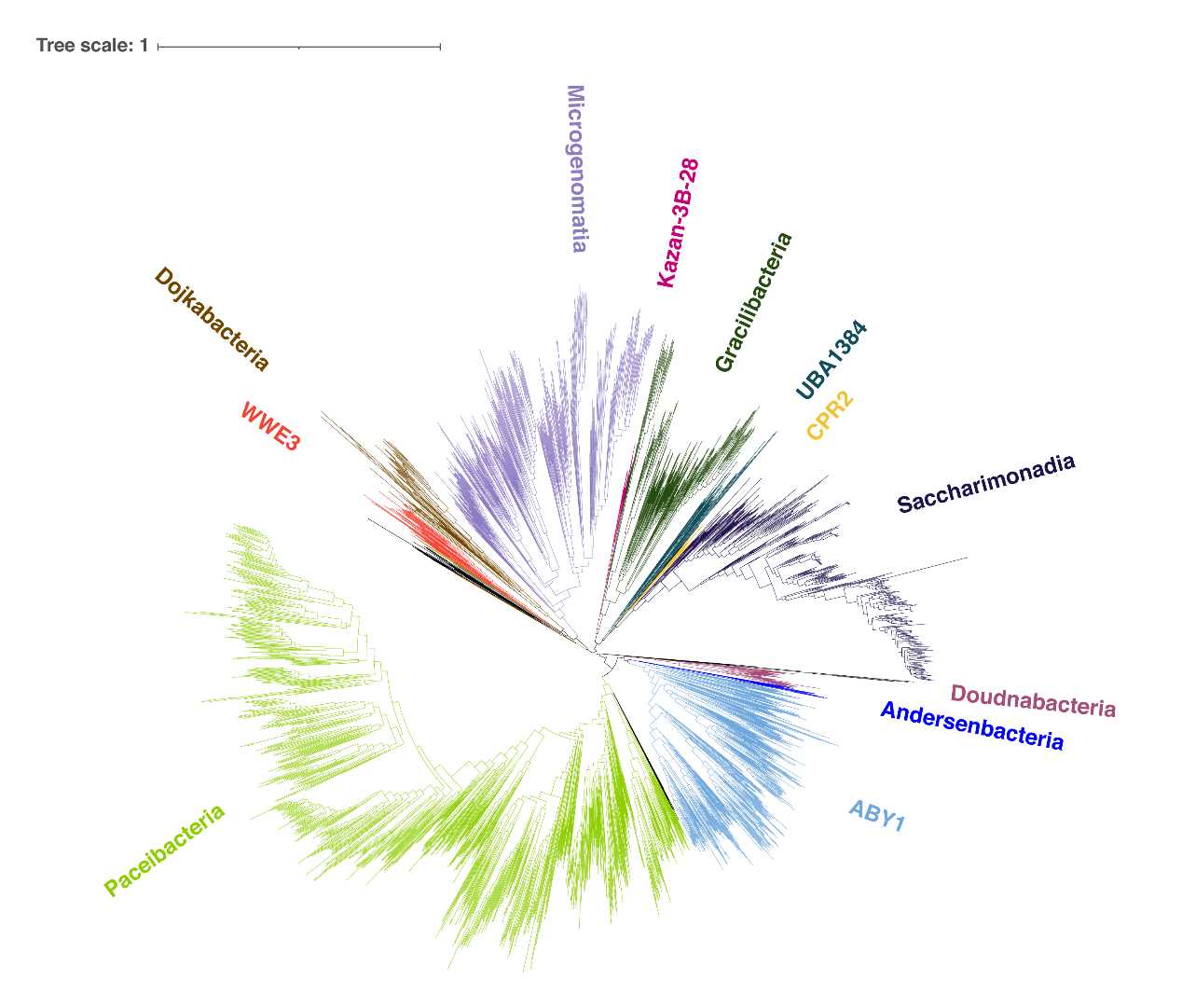
**

**Fig S3.** **Phylogenetic tree of the CPR based on 15 concatenated ribosome proteins.** Class-level lineages within the CPR are marked with different colors.


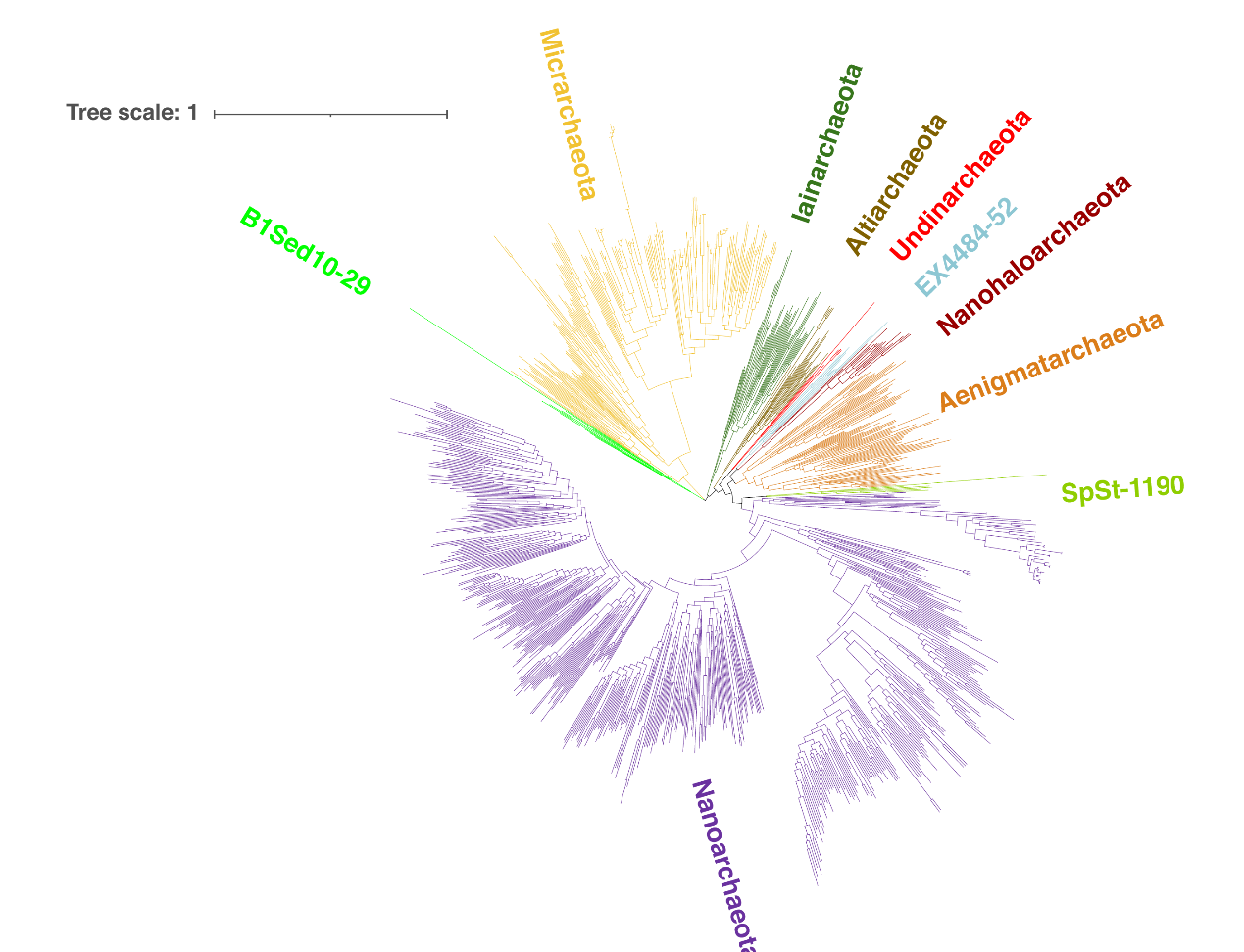


**Fig S4.** **Phylogenetic tree of the DPANN based on 14 concatenated ribosome proteins.** Phylum-level lineages within the DPANN are marked with different colors.


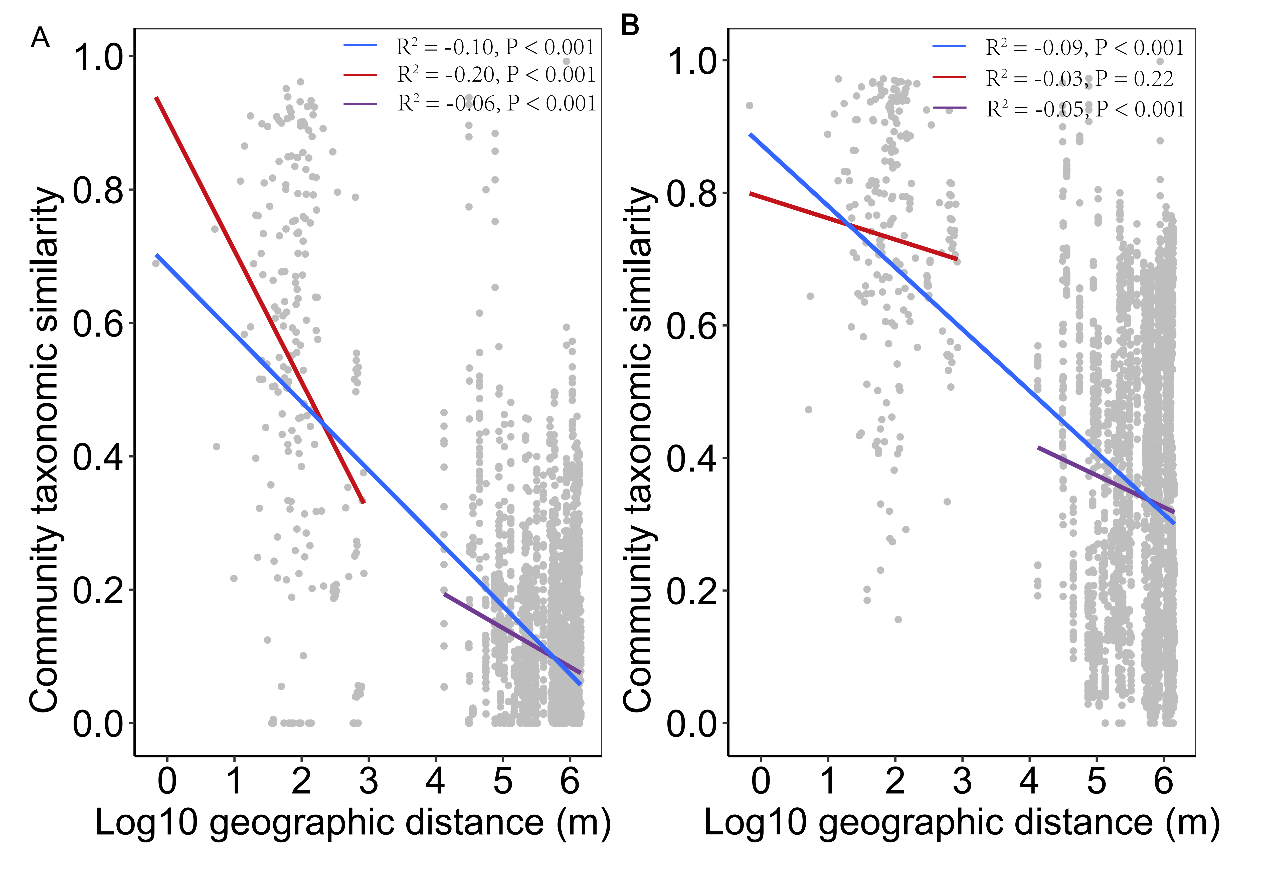


**Fig S5.** **Distance-decay relationships (DDRs) based on Bray-Curtis similarity (1 - dissimilarity) of CPR (A) and DPANN (B) taxonomic composition.** The blue line represents the overall least-squares linear regression across spatial scales. Red and purple lines represent separate regressions within mines at distances ≤ 1 km and between mines at distances > 1 km, respectively. Best fit line and adjusted R2 value shown for each color.


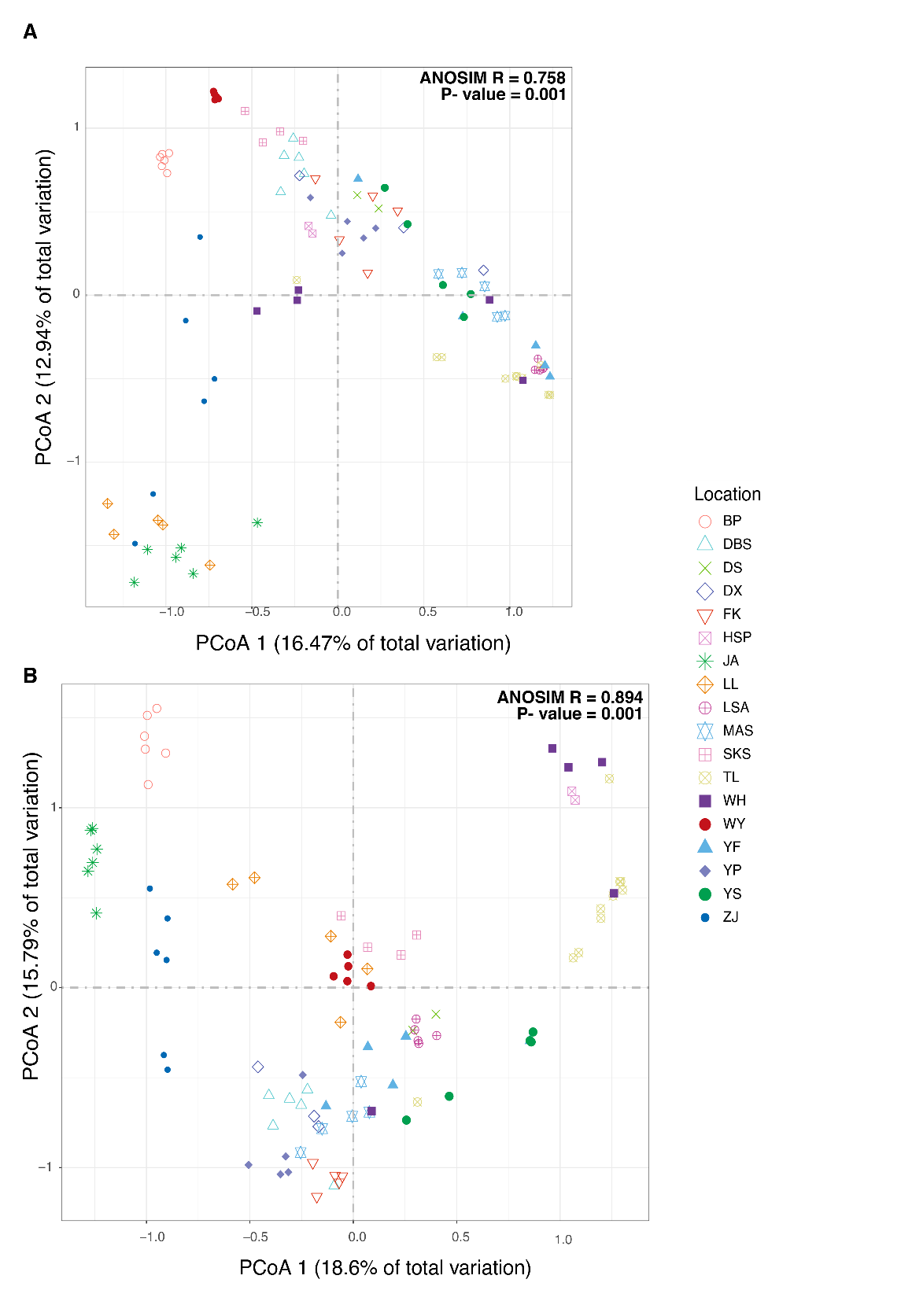


**Fig S6.** **Principal coordinate analysis (PCoA) and ANOSIM tests of CPR (A) and DPANN (B) taxonomic composition.** Dots of different colors represent different mining areas.


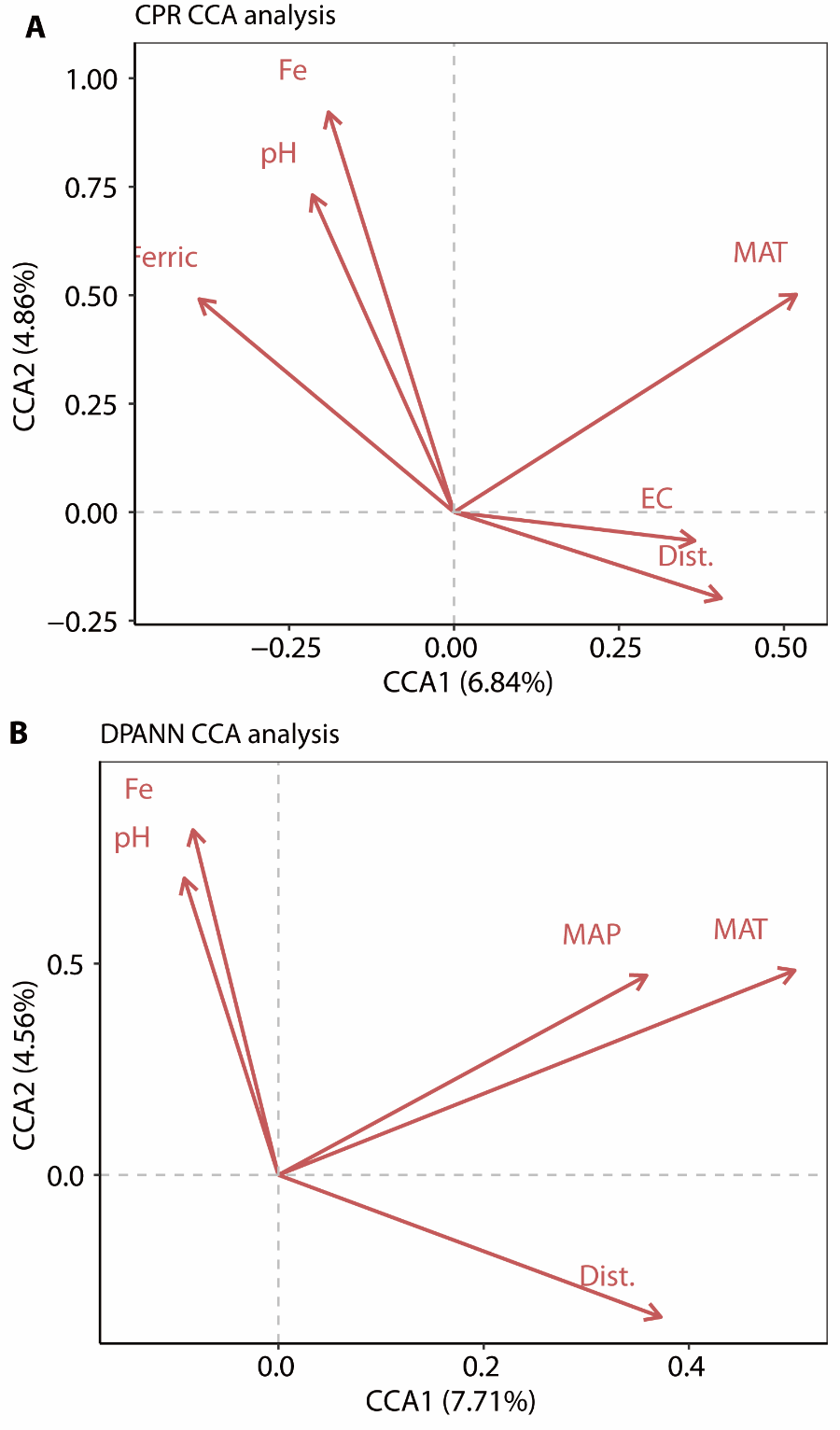


**Fig S7.** **Canonical correlation analysis of the distribution pattern of CPR (A) and DPANN (B).** The horizontal and vertical coordinates represent the first canonical variate correlation and the second canonical variate correlation, respectively.


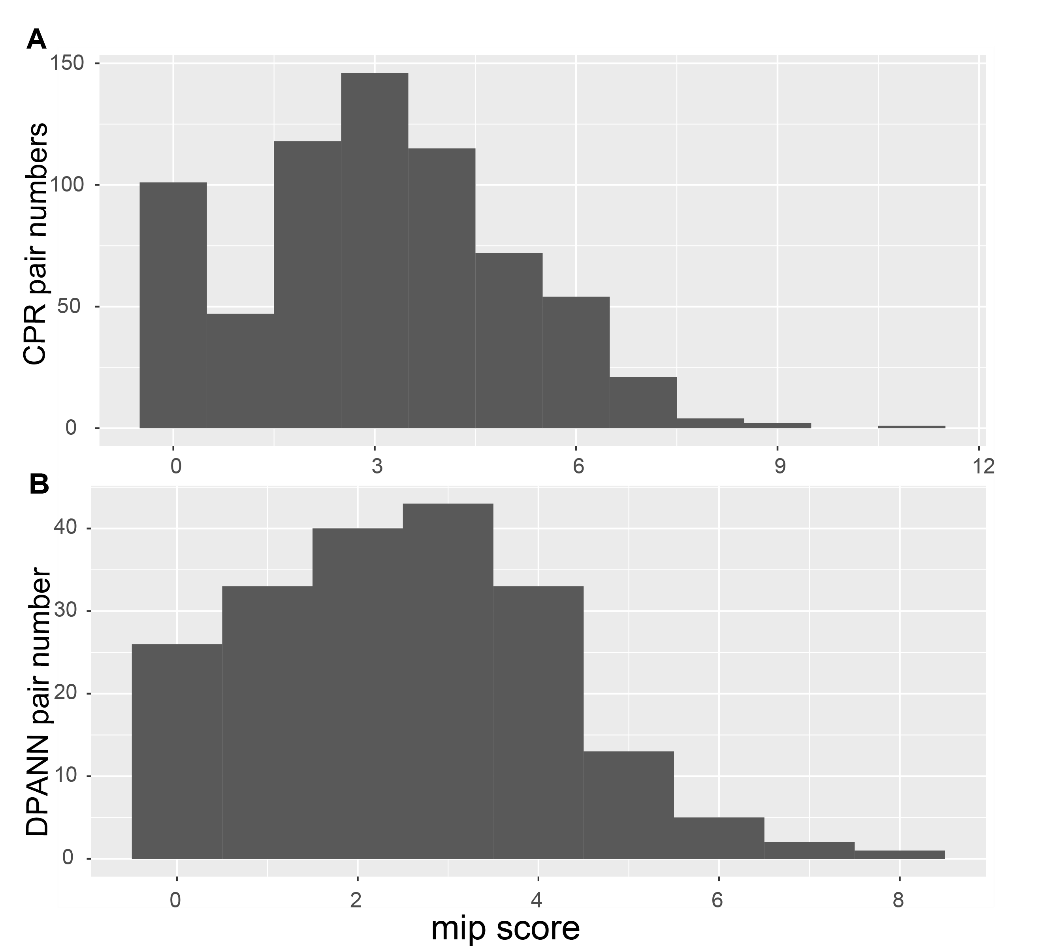


**Fig S8.** **metabolic interaction potential(MIP) score distribution diagram of CPR (A) and DPANN(B).**


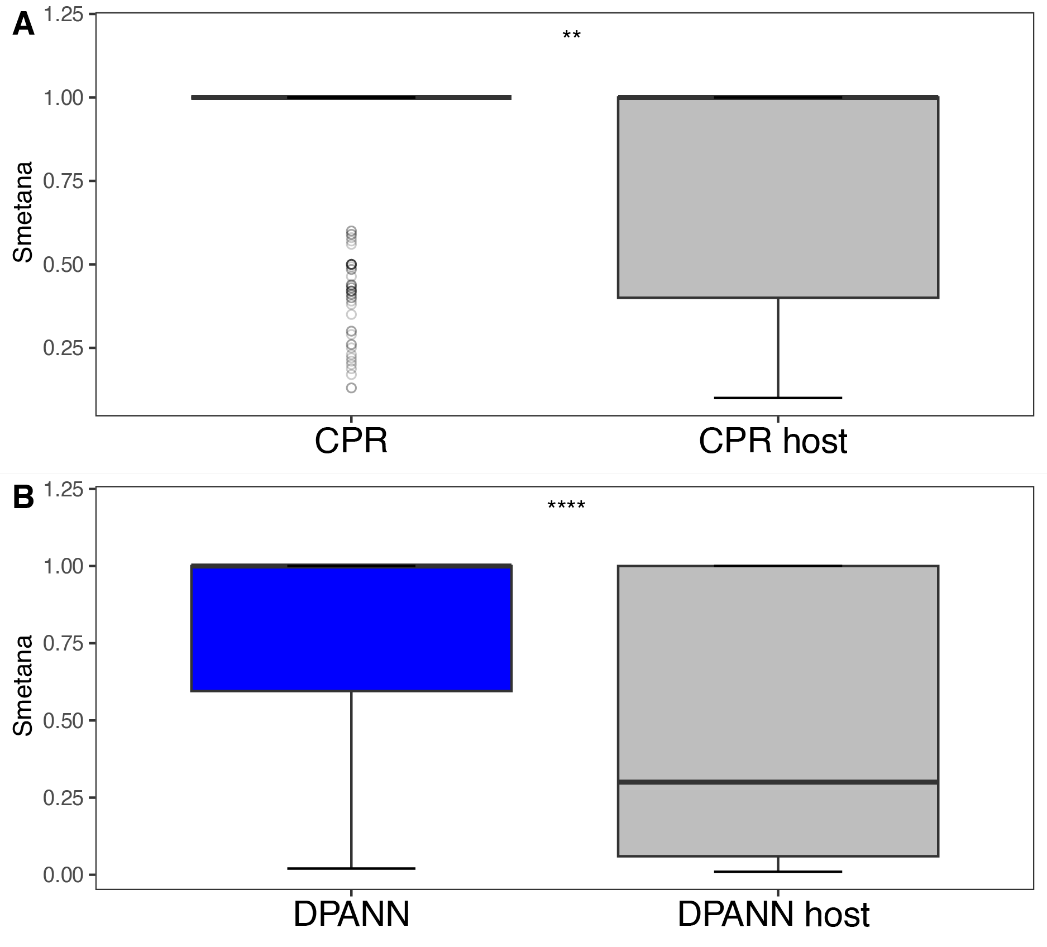


**Fig S9.The smetana scores were compared using the Wilcoxon rank sum test when CPR (A)/DPANN (B) or their hosts as receiver.** The smetana scores represent a measure of certainty on metabolic exchange. ns 0.05 < *P*, * 0.01 < *P* < 0.05, ** 0.001 < *P* < 0.01, *** 0.0001 < *P* < 0.001 and **** *P* < 0.0001.
